# International Clinical Practice Guidelines for Acute Deep Vein Thrombosis: A Comparative Review of Recommendations, Evidence Gaps, and Emerging Trends

**DOI:** 10.3390/jcm15166201

**Published:** 2026-08-11

**Authors:** Alejandro José Gonzalez-Ochoa, Juliana de Miranda Vieira, Paola Ortiz, Joana Storino, Konstantinos Kavallieros, Takaya Murayama, Nicole Marie Yuja Valle, Joana Margarida Magalhães Ferreira, Windsor Ting

**Affiliations:** 1 Department of Vascular Surgery, Centro Médico del Noroeste, San Luis Río Colorado 83449, Mexico; 2 Department of Angiology, Pedro Ernesto University Hospital, State University of Rio de Janeiro (UERJ), Rio de Janeiro 20550, Brazil; 3 Department of Vascular Surgery-Phlebology, Hospital Pasteur, Montevideo 11400, Uruguay; 4 Department of Vascular Surgery, Rede Mater Dei de Saúde, Belo Horizonte 30190, Brazil; 5 Nottingham University Hospitals NHS Trust, Nottingham NG7 2UH, UK; 6 Kannai Medical Clinic, Yokohama 231-0005, Japan; 7 Hospital Centro Urológico Hondureño, Tegucigalpa 11101, Honduras; nicoleyuja@gmail.com; 8 Department of Angiology and Vascular Surgery, São João University Hospital, 4200-319 Porto, Portugal; 9 Division of Vascular Surgery, Department of Surgery, Icahn School of Medicine at Mount Sinai, New York, NY 10029, USA

**Keywords:** acute deep vein thrombosis, clinical practice guidelines, venous thromboembolism, anticoagulation, direct oral anticoagulants, D-dimer, isolated distal deep vein thrombosis

## Abstract

Acute deep vein thrombosis (DVT) continues to represent a significant global contributor to morbidity and mortality. Although multiple international societies have published evidence-based clinical practice guidelines, important differences persist. This structured narrative review compares contemporary international guidance to identify areas of consensus, methodological divergence, evidence gaps, and priorities for future research. Recommendations were compared across diagnostic evaluation, anticoagulant selection, treatment duration, outpatient management, compression therapy, endovascular intervention, and special populations. Broad agreement was identified regarding clinical pretest probability assessment, D-dimer testing, compression ultrasonography, and direct oral anticoagulants as first-line therapy for most eligible patients with acute DVT. Clinically important differences remain, however, in the management of isolated distal DVT, anticoagulation duration, catheter-directed interventions, compression therapy, and selected high-risk populations. These discrepancies primarily reflect differences in publication timing, methodological frameworks, interpretation of emerging evidence, and healthcare-system context rather than fundamentally conflicting evidence. Contemporary international guidance therefore demonstrates substantial convergence on the core principles of acute DVT management while continuing to differ in areas supported by limited or evolving evidence. Future harmonization will require high-quality randomized trials, broader international collaboration, and the integration of precision medicine, validated risk-prediction models, biomarkers, and emerging anticoagulant strategies into guideline development.

## 1. Introduction

Deep vein thrombosis (DVT) remains a major global health challenge and a leading cause of preventable morbidity, mortality, and healthcare expenditure. As one of the principal manifestations of venous thromboembolism (VTE), acute DVT is associated with potentially fatal pulmonary embolism, recurrent thromboembolic events, post-thrombotic syndrome (PTS), chronic venous insufficiency, and long-term impairment in quality of life. Prompt diagnosis and timely initiation of evidence-based therapy are therefore essential to reduce complications, prevent recurrence, and improve both short- and long-term clinical outcomes [1,2].

During the past decade, substantial advances in diagnostic imaging, anticoagulant therapy, catheter-based interventions, and risk stratification have transformed the management of acute DVT. In parallel, several international professional societies and multidisciplinary expert panels have published updated clinical practice guidelines and consensus documents intended to standardize care and facilitate evidence-based clinical decision-making. Although these publications share common therapeutic objectives, important differences remain regarding diagnostic algorithms, D-dimer utilization, imaging strategies, anticoagulant selection, treatment duration, compression therapy, outpatient management, and indications for endovascular intervention [3,4,5,6,7,8,9,10,11,12,13,14,15,16,17].

These differences should not necessarily be interpreted as conflicting recommendations. Rather, they frequently reflect variations in publication timing, evidence appraisal methodology, healthcare-system organization, resource availability, regional epidemiology, regulatory considerations, and specialty-specific perspectives. Consequently, recommendations that appear discordant may instead represent context-specific adaptations of the same underlying evidence. Distinguishing genuine disagreement from methodological or contextual variation is essential for clinicians seeking to apply international recommendations appropriately within their local healthcare environments.

Although previous publications have summarized individual clinical practice guidelines [8,9,10], few have comprehensively compared contemporary international guidelines together with recently published multidisciplinary consensus statements while examining the reasons underlying differences among recommendations. Such comparative analyses are increasingly important as evidence continues to evolve rapidly and clinicians must integrate recommendations originating from multiple organizations.

Accordingly, this review provides a structured comparison of contemporary international clinical practice guidelines and consensus documents for the diagnosis and management of acute DVT. It emphasizes areas of consensus, methodological divergence, unresolved controversy, evidence gaps, and emerging therapeutic developments likely to influence future guideline updates. By clarifying the reasons underlying differences among recommendations, this review aims to facilitate evidence-based clinical decision-making and inform priorities for future international guideline development.

## 2. Materials and Methods

This structured narrative review compared contemporary international clinical practice guidelines and multidisciplinary consensus documents addressing the diagnosis and management of acute lower-extremity DVT. Its primary objective was to identify areas of consensus, methodological divergence, evidence gaps, and emerging concepts likely to influence future guideline development. Given the methodological heterogeneity among guideline-producing organizations, a narrative comparative approach was considered more appropriate than quantitative synthesis.

A structured literature search was conducted through July 2026 to identify the most recent international evidence-based recommendations for the diagnosis and management of acute DVT. PubMed/MEDLINE, Embase, Scopus, and Google Scholar were searched electronically, supplemented by manual searches of the official websites of major international professional societies in thrombosis, hematology, vascular surgery, cardiovascular medicine, and venous disease. The reference lists of eligible publications were also reviewed to identify additional relevant documents.

**Figure 1:**
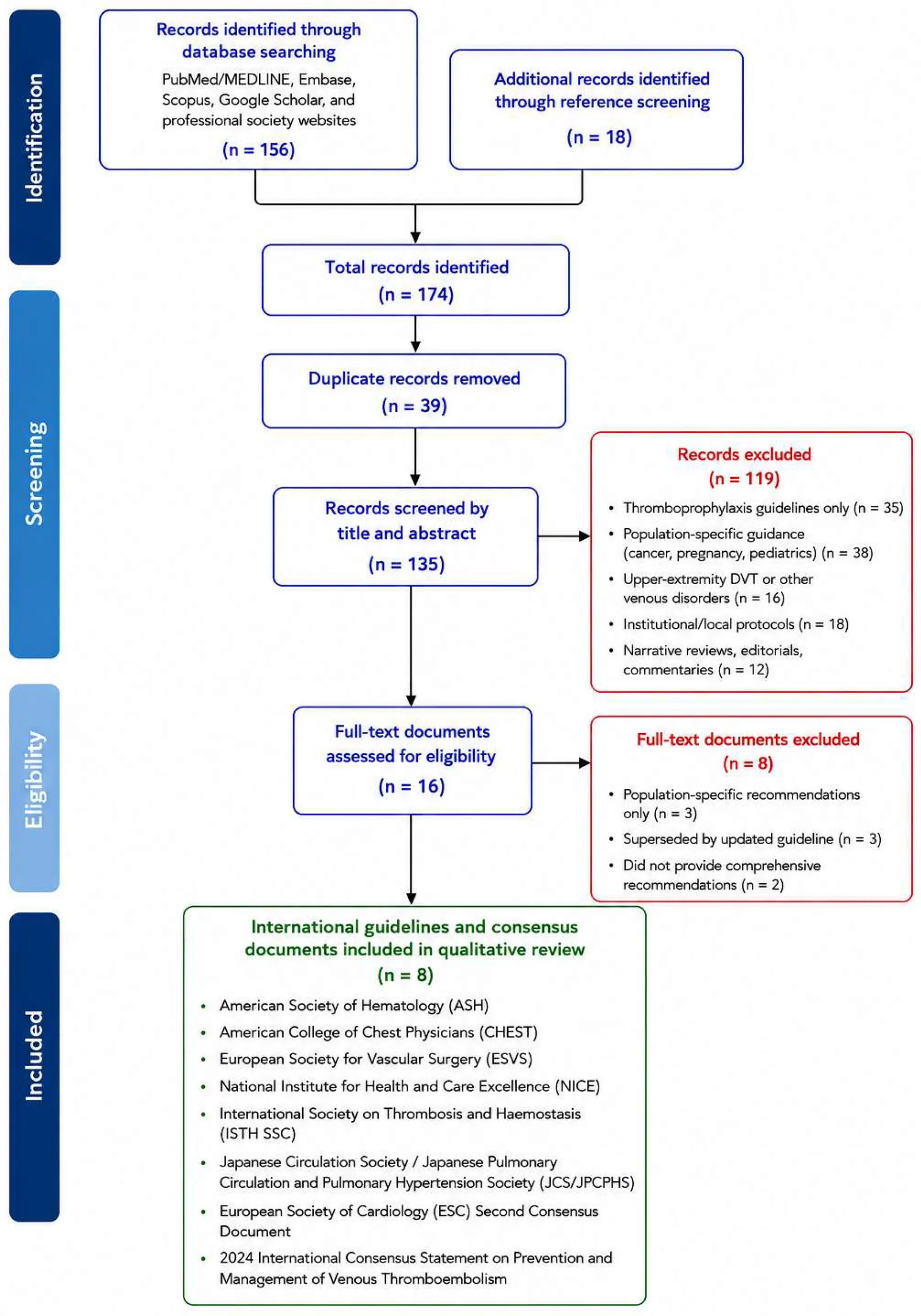
**PRISMA-inspired flow diagram**. Literature search and selection of international guidelines for the diagnosis and management of acute lower-extremity deep vein thrombosis.

Search terms included combinations of “deep vein thrombosis,” “venous thromboembolism,” “clinical practice guideline,” “guideline,” “recommendation,” “management,” “diagnosis,” and the names of relevant professional societies. The complete study selection process is summarized in Figure 1.

Documents were eligible when they met all of the following criteria: they represented the most recent version available through July 2026; provided comprehensive recommendations for the diagnosis and management of acute lower-extremity DVT; were developed by an internationally recognized professional society or a multinational, multidisciplinary expert panel; employed an explicit evidence-review methodology, including GRADE, a Class of Recommendation/Level of Evidence system, the NICE evidence framework, or another structured consensus methodology; and were intended for broad clinical application rather than a narrowly defined patient subgroup. Documents were excluded if they focused exclusively on thromboprophylaxis; addressed only a single clinical population (e.g., cancer-associated thrombosis, pregnancy, pediatric thrombosis, or upper-extremity DVT); represented institutional or local practice protocols; or had been superseded by a more recent comprehensive guideline. Selection was designed to capture geographic, methodological, healthcare-system, and specialty diversity. Accordingly, the term ‘international’ denotes the multinational and multisociety scope of the comparison and does not imply the exhaustive inclusion of all national or specialty-specific guidance.

The primary analysis included comprehensive clinical practice guidelines published by the American Society of Hematology (ASH) [3], American College of Chest Physicians (CHEST) [4], European Society for Vascular Surgery (ESVS) [5], National Institute for Health and Care Excellence (NICE) [6], and Japanese Circulation Society/Japanese Pulmonary Circulation and Pulmonary Hypertension Society (JCS/JPCPHS) [7].

Because the International Society on Thrombosis and Haemostasis (ISTH) does not publish a single comprehensive guideline dedicated to acute lower-extremity DVT, relevant Scientific and Standardization Committee (SSC) guidance documents were reviewed collectively and analyzed according to their specific clinical focus [11,12,13,14].

Two multidisciplinary international consensus documents were also included: the Second Consensus Document on Diagnosis and Management of Acute Deep Vein Thrombosis developed by the European Society of Cardiology (ESC) Working Group [15], and the Prevention and Management of Venous Thromboembolism International Consensus Statement (ICS), developed under the auspices of the European Venous Forum, North American Thrombosis Forum, International Union of Angiology, Union Internationale de Phlébologie, Cardiovascular Disease Educational and Research Trust, and Cyprus Cardiovascular Disease Educational and Research Trust [16].

Although consensus documents are methodologically distinct from formal clinical practice guidelines, they were included because of their multinational expert authorship, comprehensive scope, incorporation of recently published evidence, and growing influence on contemporary clinical practice.

Recommendations were categorized according to diagnostic evaluation, anticoagulant selection, treatment duration, compression therapy, outpatient management, catheter-directed interventions, and special populations. Areas of agreement, disagreement, and evidentiary uncertainty were identified through comparative analysis. Particular attention was given to recommendations supported by low-certainty evidence, regional differences in healthcare resources, and emerging therapeutic strategies likely to influence future guideline updates.

Because guideline development frameworks differed substantially among organizations, each recommendation was interpreted within its original grading system (e.g., GRADE, Class of Recommendation/Level of Evidence, NICE recommendation wording, or structured consensus methodology). Direct comparison of recommendation strength across organizations was therefore undertaken cautiously and within the context of each document’s methodological framework.

## 3. Overview of Contemporary International Clinical Practice Guidelines and Consensus Documents for Acute Deep Vein Thrombosis

Several international professional societies and multidisciplinary expert groups have developed evidence-based clinical practice guidelines or consensus documents to support the diagnosis and management of acute DVT. Although these publications share the overarching objective of promoting evidence-based, patient-centered care, they differ in methodological development, grading systems, update strategies, healthcare context, and therapeutic emphasis. Appreciating these differences is fundamental to understanding why recommendations may vary despite being derived from largely similar bodies of evidence.

The principal documents included in this review are summarized in Table 1. Together, they represent the most comprehensive contemporary guidance available for the management of acute lower-extremity DVT and encompass perspectives from hematology, vascular surgery, cardiovascular medicine, thrombosis, and multidisciplinary expert panels.

## 4. Guideline Comparison: Areas of Consensus, Divergence, and Evidence Gaps

Contemporary international guidelines demonstrate substantial convergence on the fundamental principles of acute DVT management. Nevertheless, several clinically important areas remain characterized by genuine uncertainty, highlighting priorities for future research and guideline development. The comparative findings and corresponding recommendation strengths are summarized in Table 2.

### 4.1. Diagnostic Evaluation

Diagnostic evaluation is an area of strong international consensus. Guidelines recommend combining clinical pretest probability, D-dimer testing, and compression ultrasonography to improve diagnostic accuracy and reduce unnecessary imaging and anticoagulation. NICE favors a structured two-level Wells score with defined diagnostic timelines, whereas ASH, CHEST, ESVS, and the International Consensus Statement allow greater adaptation to local prevalence and practice.

Compression ultrasonography is universally endorsed as the first-line imaging modality, although examination strategies differ. ESVS, ISTH, and the International Consensus Statement favor whole-leg ultrasonography, whereas CHEST and NICE generally support initial proximal imaging followed by repeat examination when clinical suspicion persists. These differences primarily reflect variation in expertise, resource availability, and concern regarding the detection of isolated distal DVT of uncertain clinical significance. Computed tomography or magnetic resonance venography is reserved for suspected iliac, pelvic, or caval thrombosis, whereas invasive venography is used mainly during endovascular procedures.

### 4.2. Initial Anticoagulant Therapy

Current international guidelines recommend DOACs as the initial treatment of choice for most patients with acute proximal DVT due to their effectiveness, safety, convenience, and reduced need for laboratory monitoring. Nonetheless, LMWH, UFH, or VKAs continue to be indicated in selected situations such as pregnancy, advanced renal dysfunction, and triple-positive antiphospholipid syndrome. The management of cancer-associated thrombosis (CAT) requires greater nuance. LMWH remains a preferred option for patients with active gastrointestinal or genitourinary malignancies associated with a high risk of mucosal bleeding, clinically significant interactions with systemic anticancer therapies, severe thrombocytopenia, unstable oral intake, or advanced renal dysfunction. Recommendations for CAT should therefore distinguish tumor location, anticipated bleeding risk, concomitant medications, renal function, and hematologic status rather than present a single management strategy for all patients with malignancy.

These exceptions reinforce the need to individualize anticoagulant selection according to clinical characteristics, contraindications, and available evidence.

### 4.3. Duration of Anticoagulation

All contemporary guidelines recommend at least three months of anticoagulation for proximal DVT. Subsequent treatment should be individualized according to recurrence risk, bleeding risk, and persistent provoking factors. Although ASH, CHEST, ESVS, ESC, and the International Consensus Statement use different terminology for treatment phases, they consistently support periodic reassessment and extended anticoagulation when clinically justified. Thus, most differences are semantic rather than therapeutic. Aspirin may be considered only after anticoagulation has been discontinued in patients with unprovoked venous thromboembolism who are not candidates for indefinite anticoagulation. Importantly, aspirin is substantially less effective than continued anticoagulant therapy and should not be regarded as an equivalent alternative.

### 4.4. Compression Therapy

Compression therapy remains one of the most debated aspects of acute DVT management. ASH and CHEST advise against routine stockings solely to prevent PTS, whereas ESVS, ESC, JCS/JPCPHS, and ICS support early compression for symptom relief and selective long-term use. These positions are largely complementary because they evaluate different outcomes: prevention of PTS versus improvement in pain, edema, function, and quality of life.

### 4.5. Outpatient Management and Endovascular Intervention

There is broad international agreement that most clinically stable patients with uncomplicated DVT can be treated safely as outpatients when anticoagulation, education, and reliable follow-up are available. Hospitalization is generally reserved for limb-threatening thrombosis, severe symptoms, high bleeding risk, major comorbidities, or inadequate social support. Catheter-directed thrombolysis and mechanical thrombectomy are not routinely recommended but may be considered in carefully selected patients with severe iliofemoral DVT. Differences among guidelines largely reflect differing priorities: hematology-focused guidance emphasizes the prevention of recurrence and bleeding, whereas vascular guidance places greater emphasis on symptom relief, venous patency, and prevention of post-thrombotic morbidity.

### 4.6. Special Populations

The management of special populations represents one of the greatest sources of heterogeneity among contemporary guidance documents; detailed disease-specific recommendations are beyond the scope of this review.

The ISTH provides the most comprehensive disease-specific guidance addressing obesity, pregnancy, inherited thrombophilia, laboratory monitoring, and cancer-associated thrombosis. In contrast, the ICS and the ESC integrate recently published evidence across a broader range of complex clinical scenarios while incorporating multidisciplinary perspectives that extend beyond traditional hematology practice.

Most observed differences reflect variation in scope and healthcare context rather than fundamentally opposing interpretations of the available evidence.

### 4.7. Evidence Gaps

Despite substantial advances in the management of acute DVT, several clinically important questions remain unresolved.

Optimal management of isolated distal DVT continues to be debated, particularly regarding the balance between immediate anticoagulation and serial ultrasonographic surveillance in carefully selected low-risk patients. Similarly, the optimal duration of anticoagulation after completion of the initial treatment phase remains uncertain, as current recommendations continue to rely primarily on broad clinical categories rather than individualized prediction of thrombotic and bleeding risk.

Although numerous biomarkers and clinical prediction models have shown promise for risk stratification, prospective evidence demonstrating improved patient outcomes remains insufficient [18,19,20,21].

Additional uncertainty persists regarding catheter-directed thrombolysis, mechanical thrombectomy, and the identification of patients most likely to derive durable clinical benefit without unacceptable bleeding risk. Likewise, management of cancer-associated thrombosis, severe obesity, advanced renal dysfunction, antiphospholipid syndrome, pregnancy, inherited thrombophilia, and frailty continues to evolve as new evidence emerges. Consequently, these areas remain high priorities for future randomized trials and international guideline harmonization.

## 5. Practical Implications for Clinical Decision-Making When Guidelines Differ

When recommendations differ, clinicians should consider whether newer evidence became available after publication of earlier guidelines and whether that evidence is sufficiently robust to justify modifying established practice. Differences should also be interpreted within the methodological framework used by each organization.

Clinical decision-making should remain individualized. Factors including thrombus location, symptom severity, bleeding risk, provoking factors, active malignancy, pregnancy, renal function, frailty, anticipated life expectancy, and patient preferences frequently influence management decisions more than minor differences among guideline recommendations. Consequently, shared decision-making remains essential, particularly when evidence is limited or multiple evidence-based treatment options are acceptable.

Healthcare context must also be considered. Recommendations developed within highly resourced healthcare systems may not be directly applicable to regions where rapid diagnostic imaging, specialist follow-up, advanced endovascular therapies, or unrestricted access to direct oral anticoagulants is limited. In such settings, appropriate adaptation of international recommendations may improve both feasibility and equity without compromising evidence-based care.

Finally, recommendations supported primarily by expert opinion or low-certainty evidence should be regarded as areas of ongoing scientific uncertainty rather than definitive standards of care. These situations require periodic reassessment as new evidence emerges and should stimulate participation in collaborative clinical research designed to address persistent knowledge gaps.

## 6. Future Directions

Future international DVT guidelines are expected to shift toward personalized, data-driven management by integrating clinical characteristics, biomarkers, advanced imaging, bleeding risk, and emerging therapies [18,22,23,24,25]. Although heparins and VKA may remain appropriate for selected high-risk populations, including patients with advanced renal impairment, active cancer, or pregnancy, ongoing clinical trials are expected to further clarify the role of DOACs in these groups [17].

One of the most promising areas of development is the incorporation of validated biomarkers and clinical prediction models into individualized anticoagulation strategies. Biomarkers, including D-dimer, LDL, factor VIII, residual venous obstruction, thrombin-generation measures, inflammatory markers, and indicators of endothelial dysfunction, may improve the prediction of recurrent venous thromboembolism and major bleeding [23]. Similarly, validated clinical prediction models, including HERDOO2, Vienna, DASH, and VTE-BLEED [19,21,24,25], provide structured approaches to balancing the benefits and risks of extended anticoagulation. However, prospective evidence demonstrating improved clinical outcomes remains necessary before these tools can be routinely incorporated into international guideline recommendations.

Factor XI and factor XIa inhibitors represent an important therapeutic advance currently under investigation. By selectively targeting the intrinsic coagulation pathway while largely preserving physiological hemostasis, agents including abelacimab, milvexian, asundexian, and fesomersen have demonstrated encouraging antithrombotic effects with comparatively low rates of clinically relevant bleeding in selected patient populations [26,27,28,29,30]. Although these findings support the concept that factor XI contributes more substantially to pathological thrombosis than to normal hemostasis, evidence specific to acute DVT and long-term secondary prevention remains limited [26,30]. These agents should therefore remain investigational until adequately powered phase III trials establish their efficacy, safety, and cost-effectiveness across diverse patient populations [26,30].

Artificial intelligence (AI) is also expected to play an increasingly important role in the management of venous thromboembolism. Potential applications include prediction of recurrent thrombosis and bleeding, automated interpretation of diagnostic imaging, electronic health record–based case identification, individualized estimation of treatment benefit, and clinical decision support through integration of multiple patient-specific variables [31,32,33]. Nevertheless, most currently available AI models have been developed using retrospective datasets and have not undergone rigorous prospective external validation. Concerns regarding algorithm transparency, bias, interoperability, data quality, and regulatory oversight remain important barriers to widespread implementation in routine clinical practice [31,32,33,34].

Future guideline development should also emphasize implementation science, particularly in low- and middle-income countries where access to diagnostic imaging, anticoagulants, and endovascular therapies may differ substantially from that assumed in many existing recommendations. Development of resource-sensitive diagnostic algorithms and treatment pathways will be essential to improve the global applicability of evidence-based care [35].

Ultimately, future international guidelines are expected to evolve toward precision medicine frameworks. Such an approach has the potential to optimize treatment duration, minimize bleeding complications, reduce recurrent thromboembolism, and improve the overall net clinical benefit of anticoagulant therapy while promoting greater international harmonization of clinical practice [30,36].

## 7. Discussion

This comparative review demonstrates that contemporary international clinical practice guidelines and multidisciplinary consensus statements are characterized by substantially greater agreement than disagreement. Where recommendations diverge, the underlying explanations are often multifactorial.

One of the most important determinants of variation is the timing of guideline publication. In isolated distal DVT, the CACTUS trial (2016) [37] found no statistically significant reduction in proximal extension or symptomatic VTE but reported increased bleeding, thereby supporting cautious surveillance strategies. The RIDTS trial (2022) [38], however, found that extending rivaroxaban therapy to three months reduced recurrent VTE, predominantly recurrent distal DVT, without increasing major bleeding. RIDTS postdated ASH 2020, NICE 2020, ESVS 2021, and CHEST 2021 and therefore could not have informed their original recommendations. Its availability to the ICS 2024 panel may have contributed to the stronger recommendation for three months of anticoagulation in symptomatic calf DVT. Although RIDTS does not directly establish whether every patient with low-risk distal DVT requires initial anticoagulation, the remaining disagreement should often be interpreted as reflecting the natural evolution of scientific evidence rather than inconsistency in clinical reasoning.

For catheter-directed thrombolysis and early thrombus removal, CaVenT suggested a reduction in PTS following catheter-directed thrombolysis [39]. ATTRACT (2017) [40] found no reduction in overall PTS after pharmacomechanical thrombolysis for proximal DVT and reported an increased risk of early major bleeding, although moderate-to-severe PTS and symptom severity were reduced. CAVA (online 2019; print 2020) [41] did not demonstrate a clear primary benefit of ultrasound-accelerated thrombolysis in iliofemoral DVT. Because these trials were available to the 2020–2021 guideline panels, publication timing alone does not explain the divergence. Instead, differing recommendations reflect the relative emphasis placed on overall PTS incidence versus PTS severity and early symptom relief, together with genuinely different interpretations of mixed evidence, specialty perspectives, and healthcare-system capacity.

Variation in recommendation strength also reflects differences in the methodologies used to develop international guidance. Organizations using the GRADE framework, including ASH and CHEST, explicitly distinguish certainty of evidence from recommendation strength while placing considerable emphasis on patient values, resource utilization, feasibility, and the balance between desirable and undesirable outcomes. ESVS and JCS/JPCPHS employ the traditional Class of Recommendation/Level of Evidence system, which classifies recommendations according to clinical usefulness and supporting evidence. NICE additionally integrates health-economic analyses and implementation considerations into recommendation development without assigning explicit recommendation grades. In contrast, multidisciplinary consensus statements combine evidence review with structured expert consensus to address clinical questions for which randomized evidence remains incomplete. Each recommendation should therefore be interpreted within the methodological framework through which it was developed.

Organizational priorities provide another explanation. Hematology societies emphasize prevention of recurrent venous thromboembolism, anticoagulant optimization, and bleeding reduction. Vascular and cardiovascular organizations place greater emphasis on venous anatomy, imaging, symptom relief, preservation of venous function, prevention of post-thrombotic syndrome, and selective endovascular intervention. These perspectives are often complementary rather than contradictory. Healthcare context further shapes recommendations: publicly funded systems may prioritize cost-effectiveness and service organization [35], whereas international statements may offer broader flexibility. Asian guidance may also account more explicitly for ethnic differences in thrombotic and bleeding risk and regional resource constraints [42].

Greater collaboration among professional societies could facilitate standardized evidence appraisal, harmonized definitions of clinical outcomes, coordinated prioritization of research questions, and more consistent approaches to grading recommendations [43]. At the same time, sufficient flexibility should be preserved to allow adaptation to regional healthcare systems, resource availability, and population characteristics.

Emerging developments, including precision medicine, validated risk-prediction models, biomarkers [44], artificial intelligence, and novel anticoagulant therapies such as factor XIa inhibitors, are likely to influence future iterations of international guidelines. Incorporating these advances into evidence-based recommendations may improve individualized treatment selection while reducing unwarranted variation in clinical practice.

This review is strengthened by its comprehensive comparison, contextual interpretation, and inclusion of recent international guidance. Its principal limitations are the use of a structured narrative approach without quantitative synthesis, the limited comparability of grading systems, the continuing evolution of recommendations, and regional differences that may restrict universal applicability. Despite these limitations, the analysis provides a clinically useful framework for interpreting apparent disagreements and identifying priorities for future research.

## 8. Conclusions

Contemporary international guidelines show broad agreement on the diagnosis and treatment of acute DVT. Strong consensus supports clinical probability assessment, D-dimer testing, compression ultrasonography, and prompt anticoagulation with direct oral anticoagulants for most eligible patients. Differences remain in the management of isolated distal DVT, compression therapy, catheter-directed interventions, extended anticoagulation, and special populations. These variations generally reflect evolving evidence, methodological frameworks, specialty perspectives, and healthcare-system contexts rather than conflicting scientific conclusions.

Rather than regarding differing recommendations as contradictory, clinicians should interpret them within their original context and individualize treatment according to thrombotic and bleeding risks, comorbidities, patient preferences, and available resources.

## Figures and Tables

**Table 1:** **Framework of International Guidelines and Consensus Statements for Acute DVT.** Comparison of publication year, methodological framework, grading system, update status, and scope.

Guideline (Publication Year)	Methodological Framework	Grading System	Update Status	Scope
ASH (2020) [3]	Systematic reviews; explicit assessment of benefits, harms, certainty, values, resources, equity, acceptability, and feasibility.	GRADE. Recommendations classified as strong or conditional; certainty rated high, moderate, low, or very low.	Comprehensive de novo guideline within the ASH VTE guideline series; not labeled as an update. Separate ASH cancer guideline published in 2021.	Initial management, primary treatment, and secondary prevention of DVT/PE in patients without cancer; anticoagulant selection, duration, thrombolysis, IVC filters, compression, and recurrent VTE.
CHEST (2021; 2024 compendium) [4]	PICO-based evidence review under CHEST guideline methodology; selected questions from prior editions reassessed, with new questions added.	CHEST grading: strong or weak/conditional recommendation according to certainty/quality of evidence; some ungraded consensus-based statements.	Partial/selective second update of the 2016 first update. 29 statements total. The 2024 compendium consolidates 2012–2021 guidance.	Antithrombotic management from initiation through treatment and extended phases; distal DVT, cancer-associated thrombosis, cerebral venous thrombosis, PTS, and selected special situations.
ISTH (Multiple key DVT-related updates 2018–2022) [11,12,13,14]	SSC expert guidance addressing narrowly defined populations or management problems; evidence review and expert consensus vary by statement.	No single uniform grading system. Many provide ungraded guidance statements; certainty and wording depend on the individual document.	Focused and topic-specific rather than a comprehensive acute-DVT guideline. Some documents are explicitly updated communications.	Special populations and technical issues, including cancer-associated VTE, extreme obesity, bariatric surgery, COVID-19, laboratory monitoring, and other anticoagulation scenarios.
ESC (2022 online 2021) [15]	Updated narrative appraisal of new evidence by the ESC Working Groups; developed as a companion to the 2019 ESC pulmonary embolism guideline.	No formal GRADE or ESC class/level grading grid. Consensus and key messages use directive wording and are identified as new, revised, unchanged, or deleted relative to the previous document.	Comprehensive second consensus update of the 2017 document, with revisions addressing extended anticoagulation, cancer, PTS, bleeding reversal, pregnancy, and emerging evidence.	Whole spectrum of acute DVT: risk factors, diagnostic pathways, initial and first 3-month treatment, extended management, thrombus removal, vena cava filters, compression and PTS, UEDVT, cancer, pregnancy, and thrombophilia.
ESVS (2021) [5]	Structured evidence review, predefined clinical questions, consensus formulation, external peer review, Guidelines Committee oversight, and patient/public review of lay summaries.	European-style classes of recommendation (I, IIa, IIb, III) and levels of evidence (A, B, C), supplemented by expert consensus where evidence is limited.	Comprehensive first ESVS guideline devoted to venous thrombosis; a full guideline rather than a narrow update. A new revision is currently in development.	Diagnosis and management of lower- and upper-extremity DVT, anticoagulation, distal DVT, early thrombus removal, iliac stenting, IVC filters, unusual-site thrombosis, and special populations.
NICE (2020; updated 2023) [6]	NICE guideline-development manual; clinical and economic evidence reviews, PICO questions, consideration of cost-effectiveness, equality, implementation, and stakeholder consultation.	Modified GRADE approach; evidence certainty and committee interpretation support directive wording such as offer, consider, and do not offer. Recommendations are not presented as a simple class/level grid.	NG158 replaced CG144 in 2020; partial update in August 2023. It is a full replacement guideline with subsequent targeted maintenance.	Adult diagnosis and management of suspected or confirmed DVT/PE, anticoagulant choice and duration, outpatient pathways, cancer investigation, thrombophilia testing, and service delivery in the NHS.
JCS/JPCPHS (2025 English publication 2026) [7]	Japanese joint-society guideline integrating systematic evidence appraisal, expert consensus, local epidemiology, approved dosing, and Japanese clinical-practice considerations.	Classes of recommendation (I, IIa, IIb, III) and levels of evidence; consensus level is used where evidence is limited, consistent with JCS methodology.	Full revision, released in March 2025, updating the prior guideline after approximately eight years; expanded and reorganized coverage of VTE and pulmonary hypertension.	PE, DVT, and pulmonary hypertension; diagnosis, risk assessment, anticoagulation, intervention, compression, prevention, follow-up, and population-specific Japanese considerations.
ICS (2024) [15]	Section-based narrative evidence review developed by an international faculty; recommendations formulated from scientific evidence and expert debate under multiple venous and thrombosis organizations.	Levels of evidence (high, moderate, low) paired with recommendation strength (strong, moderate, weak). The approach is evidence-informed but is not a formal GRADE process.	Comprehensive full update of the 2013 international consensus statement; developed online during 2023 and published as a 2024 supplement.	Broad prevention, diagnosis, and management of VTE across 26 sections: medical and surgical prophylaxis, DVT/PE diagnosis and treatment, cancer, filters, thrombus removal, PTS, COVID-19, and special situations.

DVT, deep vein thrombosis; COVID-19, COronaVIrus Disease of 2019; ESC, European Society of Cardiology; ESVS, European Society for Vascular Surgery; GRADE, Grading of Recommendations Assessment, Development and Evaluation; ISTH, International Society on Thrombosis and Haemostasis; JCS/JPCPHS, Japanese Circulation Society/Japanese Pulmonary Circulation and Pulmonary Hypertension Society; IVC, inferior vena cava; NICE, National Institute for Health and Care Excellence; PE, pulmonary embolism; ICS, International Consensus Statement; PTS, post-thrombotic syndrome; SSC, Scientific and Standardization Committee; VTE, venous thromboembolism.

**Table 2:** Comparison of International Guideline Convergence, Divergence, Recommendation Strength, and Evidence Gaps for Acute Deep Vein Thrombosis.

Clinical Domain	Areas of Convergence	Areas of Divergence	Typical Recommendation Strength *	Major Evidence Gaps
Diagnostic strategy	All recommend structured clinical probability assessment combined with D-dimer and compression ultrasonography.	NICE mandates a Wells-based algorithm with defined timelines; ASH emphasizes probability-adjusted pathways; ICS supports validated models without endorsing a single score.	Strong (ASH, CHEST); Class I-B (ESVS, JCS); NICE: “Offer”; ICS: High-quality evidence, strong consensus.	Validation of AI-assisted diagnostic pathways and simplified diagnostic algorithms for resource-limited settings.
D-dimer	Integrated into diagnostic algorithms for low/intermediate-risk patients.	Age-adjusted thresholds adopted inconsistently; differing recommendations for recurrent DVT and special populations.	Moderate-to-strong across organizations.	Optimal biomarker integration and standardized age-adjusted strategies.
Compression ultrasonography	Universal first-line imaging modality.	Whole-leg ultrasound favored by ESVS, ISTH and ICS; proximal ultrasound with repeat imaging favored by CHEST and NICE.	Strong/Class I.	AI-assisted image interpretation; cost-effectiveness of whole-leg imaging.
Initial anticoagulation	DOACs preferred for most patients without contraindications.	Management differs in pregnancy, APS, severe renal dysfunction, cancer, and extremes of body weight.	Strong/Class I-A.	Evidence for Factor XIa inhibitors and precision anticoagulation.
Cancer-associated thrombosis	Anticoagulation is recommended in all guidelines.	DOACs are increasingly favored; LMWH remains preferred for gastrointestinal or genitourinary malignancy, thrombocytopenia, or high bleeding risk.	Conditional to strong, depending on the population.	Immunotherapy-associated thrombosis, thrombocytopenia, individualized anticoagulant selection.
Pregnancy	LMWH remains first-line therapy.	Minimal differences among guidelines.	Strong/Class I.	Postpartum anticoagulation and future evidence regarding DOACs.
Renal impairment	UFH, LMWH, or VKAs are preferred options in severe renal disease.	Guidelines use different renal-function thresholds for DOAC therapy.	Strong.	Safety of DOACs in advanced CKD or ESRD.
Duration of anticoagulation	Minimum of 3 months for proximal DVT.	ASH emphasizes provoking factors; CHEST and ESVS provide more detailed risk-stratified continuation strategies; ICS recommends individualized reassessment.	Strong/Class I.	Biomarker-guided treatment duration and individualized prediction models.
Extended anticoagulation	Recommended for selected patients at high recurrence risk.	CHEST and ICS explicitly recommend reduced-dose DOACs; ASH favors indefinite therapy when recurrence risk predominates.	Strong.	Optimal discontinuation strategies using biomarkers and prediction models.
Isolated distal DVT	Anticoagulation for symptomatic or high-risk patients.	CHEST and ESVS permit surveillance in selected low-risk patients; ICS recommends anticoagulation for symptomatic calf DVT.	Conditional to Strong.	Identification of patients suitable for surveillance versus treatment.
Compression therapy	Accepted for symptom relief.	ASH/CHEST discourage routine use solely to prevent PTS; ESVS, JCS, ESC and ICS support early compression for symptom control.	Conditional (ASH/CHEST); Class IIa (ESVS); Moderate consensus (ICS).	Optimal pressure, duration, and patient selection.
Outpatient management	Stable patients should be treated as outpatients whenever feasible.	Minor variation according to healthcare resources.	Strong.	Telemedicine and remote monitoring strategies.
Catheter-directed thrombolysis	Reserved for carefully selected patients with iliofemoral DVT.	Hematology societies are more conservative; vascular guidelines are more supportive in selected young patients with severe symptoms.	Conditional (ASH/CHEST); Class IIa (ESVS); Moderate consensus (ICS).	Identification of patients most likely to benefit.
Mechanical thrombectomy	Reserved for selected patients only.	ESVS discusses newer devices more extensively than hematology guidelines.	Weak to moderate.	High-quality randomized trials of modern thrombectomy systems.
PTS prevention	Early anticoagulation and mobilization universally recommended.	Compression recommendations differ according to therapeutic objective.	Moderate.	Prevention strategies beyond compression therapy.
Special populations	Individualized management universally recommended.	ISTH provides the most detailed disease-specific guidance; ICS and ESC include broader multidisciplinary recommendations.	Variable according to condition.	Frailty, obesity, APS, cancer, renal dysfunction, elderly patients, recurrent DVT, and multimorbidity.
Future therapies	Recognition that precision medicine will influence future guidelines.	The ICS provides the most detailed discussion of several emerging therapies.	Consensus-based.	Factor XIa inhibitors, AI, biomarkers, implementation science, precision medicine, and living guidelines.

* Recommendation strength reflects each organization’s original grading system (GRADE, Class of Recommendation/Level of Evidence, NICE wording, or structured consensus methodology) and should not be interpreted as directly comparable across organizations. ‘Strong’ corresponds to strong recommendations within GRADE-based systems. ESVS and JCS/JPCPHS recommendations are reported using Class of Recommendation (I-III) and Level of Evidence methodology. NICE does not assign explicit recommendation grades (e.g., strong or conditional) in its published clinical guidelines. Although GRADE is used during evidence appraisal, recommendation strength is conveyed through standardized wording (e.g., offer, consider, or do not offer) rather than formal labels. ISTH does not uniformly use GRADE terminology; therefore, recommendation intensity is reported according to the original guideline framework or expert consensus. Because grading systems differ substantially among organizations, direct comparisons should be interpreted cautiously. AI, artificial intelligence; APS, antiphospholipid syndrome; DOACs, direct oral anticoagulants; DVT, deep vein thrombosis; ESC, European Society of Cardiology; ESVS, European Society for Vascular Surgery; GRADE, Grading of Recommendations Assessment, Development and Evaluation; ISTH, International Society on Thrombosis and Haemostasis; JCS/JPCPHS, Japanese Circulation Society/Japanese Pulmonary Circulation and Pulmonary Hypertension Society; LMWH, low-molecular-weight heparin; NICE, National Institute for Health and Care Excellence; ICS, International Consensus Statement; PTS, post-thrombotic syndrome; UFH, unfractionated heparin; VKA, vitamin K antagonist.

## Data Availability

No new data were created or analyzed in this study. Data sharing is not applicable to this article.
